# Unilateral Retinitis Pigmentosa, Glial Tissue Abnormality, and Microphthalmia in a Young Female Patient: A Case Report

**DOI:** 10.7759/cureus.107568

**Published:** 2026-04-23

**Authors:** Logan Bivona, Taulant Rama, Gabrielle J Wolfe, Peter J Maris, Robert Lopez

**Affiliations:** 1 Osteopathic Medicine, New York Institute of Technology College of Osteopathic Medicine, Old Westbury, USA; 2 Ophthalmology, Drexel University College of Medicine, Wyomissing, USA; 3 Ophthalmology, State University of New York Upstate Medical University College of Medicine, Syracuse, USA; 4 Ophthalmology, Columbia University Irving Medical Center, New York, USA

**Keywords:** glial tissue, inositol polyphosphate-5-phosphatase e gene, microphthalmia, retinitis pigmentosa, unilateral pigmentary retinopathy

## Abstract

This report presents a case of unilateral retinitis pigmentosa (URP) with associated symptoms and microphthalmia in the right eye of a young female patient. A 23-year-old with a previous diagnosis of URP and a constricted visual field in one eye was examined. Slit lamp examination of the right eye revealed a moderate posterior subcapsular and mild nuclear sclerotic cataract, with a clear lens in the left eye. Funduscopic examination of the right eye showed diffuse retinal pigmentary epithelium atrophy with peripheral bone spicule pigmentation, cystoid macular edema, and glial clusters protruding from the optic nerve. Left eye fundoscopy revealed unremarkable retinal findings. Fundus autofluorescence, optical coherence tomography, Humphrey visual field, and full-field electroretinogram testing indicated compatible results with our funduscopic findings of URP. Axial length (AL) measurements and anterior chamber angle assessment by gonioscopy suggested microphthalmia of the right globe. The left eye was shorter than average but within normal limits. Salivary genetic testing revealed genetically sensitized retinae carrying heterozygous variants in the INPP5E, FSCN2, RP1L1, and WFS1 genes. The patient is a carrier of a pathogenic variant of INPP5E, while the significance of the other identified variants is uncertain. This case report showcases the unique presentation of URP, glial tissue abnormality, and microphthalmia, suggesting a potentially shared genetic link between retinal pigmentary degeneration and malformation of other ocular structures. Further study is required to understand any shared genetic and/or proteomic etiology. These discoveries may aid in possible future treatment modalities for retinal cilioretinopathy disease and related disorders.

## Introduction

Retinitis pigmentosa (RP), a subform of rod-cone dystrophy, refers to a group of rare hereditary retinal degenerative disorders that typically impact both eyes symmetrically [1]. RP manifests by progressive diminishing of vision due to the deterioration of retinal photoreceptors and the neighboring retinal pigment epithelium (RPE) layer [1]. Although there may be variance in specific manifestations of RP, it canonically begins with atrophy of peripheral rod cells, resulting in the loss of peripheral visual field as well as nyctalopia [1]. As the disease progresses into the macula, deterioration of both cone and rod cells leads to a further decrease in vision and constriction of the visual field [1].

RP is commonly correlated with several ocular findings, such as cystoid macular edema (CME) and posterior subcapsular cataract (PSC) [1]. While these associated conditions may be treated through a variety of means, currently, there is no clinically proven treatment for RP [1].

RP is typically inherited through autosomal dominant (AD), autosomal recessive (AR), or X-linked patterns [2]. The frequency of inheritance in males tends to be slightly more prevalent than in females due to the X-linked inheritance pattern; otherwise, there is no sex difference in the frequency of RP in the other forms of inheritance, even if sex differences may exist in the severity of disease progression [3]. The average age of onset of RP largely depends on the specific pattern of inheritance with the AD form manifesting in the 20s, while the AR variant typically appears in the early teen years [3].

Unilateral retinitis pigmentosa (URP) is an unusual phenomenon in which RP-like symptoms appear monocularly [2]. The first indisputable case of URP was published by Pedraglia in 1865 [4]. Unilaterality presents with a frequency of approximately 5% in all RP cases, making it exceedingly rare [5]. Childhood-onset URP is rarer because, in order to confirm the diagnosis, it is necessary to rule out other pigmentary retinal diseases using the criteria specified by François and Verriest [6]: presentation of characteristic retinal changes in the impacted eye; lack of retinal degenerative signs in the healthy eye as shown in full-field electroretinogram (ffERG) and fundus studies; exclusion of autoimmune, drug-related, infectious, inflammatory, and traumatic etiologies in the diseased eye; and observation period lasting at least five years to rule out the late presentation of RP in the fellow eye.

Microphthalmia is a condition in which an eye is considered small and has anatomic malformations [7]. In order to be classified as having microphthalmia, the axial length (AL) of the globe must be two standard deviations below the mean for the patient's age, which corresponds to an AL of below 21.00 mm in the typical adult [7]. Although microphthalmia has been associated with RP in the literature, there is no established general-population incidence rate as this combination occurs in rare case reports or genetic syndromes [8-11].

## Case presentation

A 23-year-old, Caucasian female patient presented with a chief complaint of blurry vision in her right eye. She does not report any concerns about her left eye. The patient reported no ophthalmic therapeutic agents. The patient's ophthalmic history is significant for a previous diagnosis of URP in her right eye. Her monocular deficit was initially caught during a local school screening program at age seven. She was promptly referred to an ophthalmologist who referred her to a medical retinal specialist with a genetics background at an academic eye center. The diagnosis of URP was first speculated and then later confirmed after an observation period of five years and a genetic test. The patient has an otherwise unremarkable medical history. Per patient recollection, original genetic testing revealed no family history of inherited ocular diseases.

**Figure 1:**
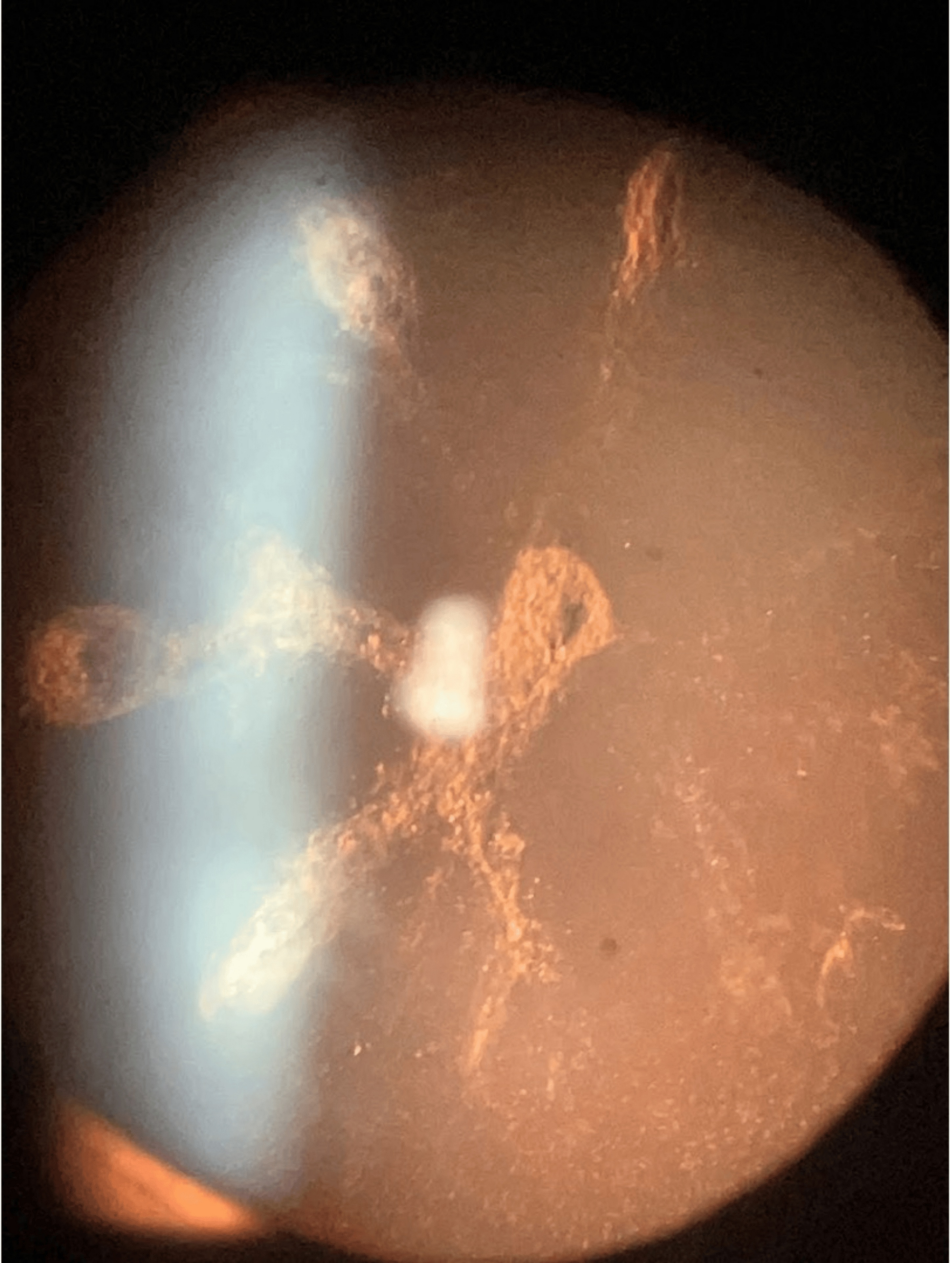
Slit lamp photograph of the right eye showing a stellate posterior subcapsular cataract

Distance visual acuities were 20/400 OD and 20/20 OS without correction. Pupillary exam revealed an afferent pupillary defect in OD. Intraocular pressure (IOP) measurements by Goldmann applanation tonometry were as follows: OD=12 mmHg and OS=13 mmHg. AL measurements per Lenstar interferometry were as follows: OD=19.84 mm and OS=22.06 mm. Gonioscopy revealed narrow anatomical angles OU. OD had a 0 configuration by Shaffer classification without any peripheral anterior synechiae [12]. OS revealed a moderately narrow angle with a grade of II and showed prominent fine iris processes throughout the chamber angle. Slit lamp examination showed a moderate, stellate PSC and a mild nuclear sclerotic (NS) cataract in OD (shown in Figure 1). The crystalline lens was clear and unremarkable in OS. While fundus examination can easily detect superficial drusen, buried optic disc drusen are more difficult to view. B-scan ultrasonography ruled out the presence of buried optic disc drusen OU and Bergmeister's papilla OU.

**Figure 2:**
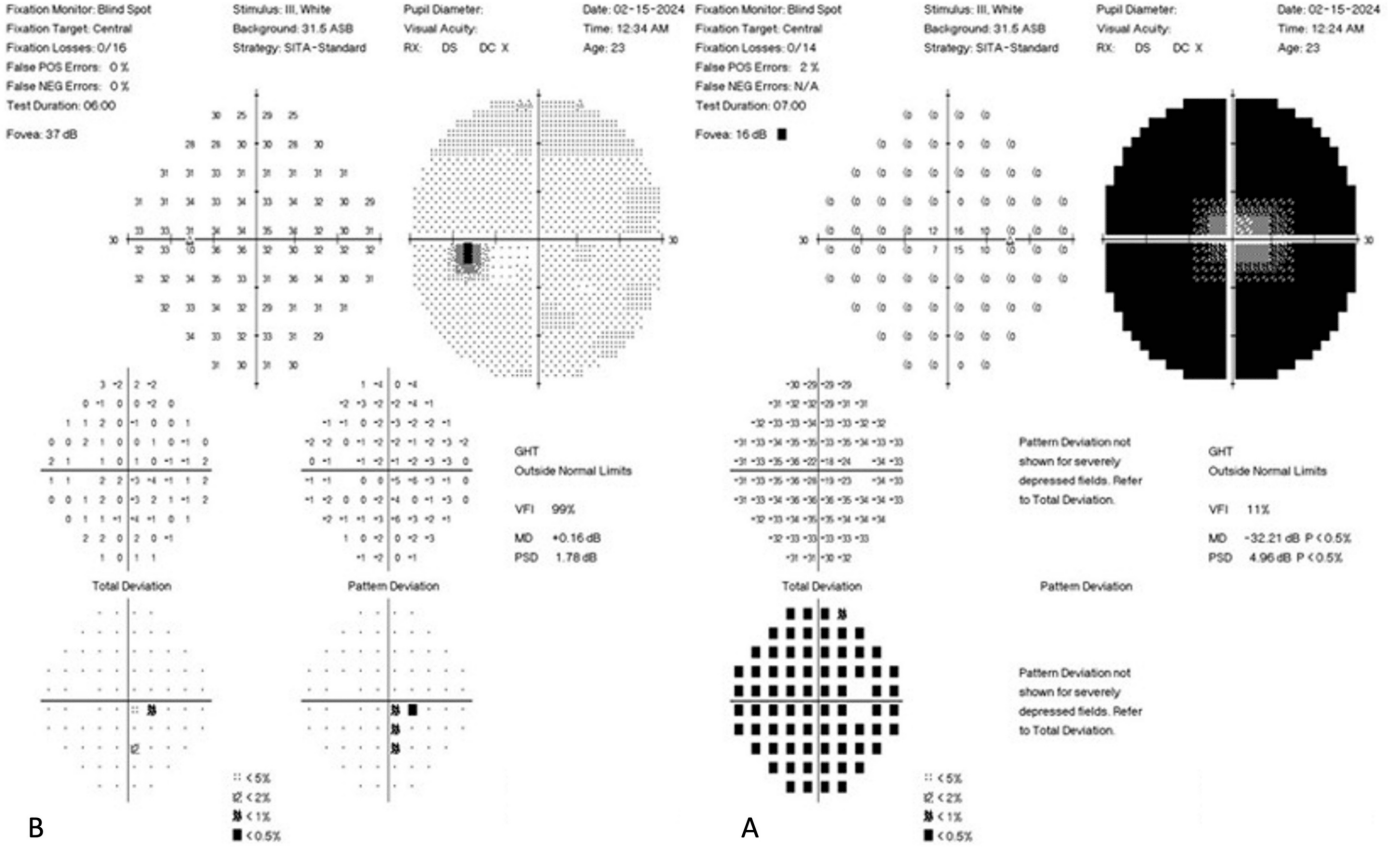
A SITA-Standard 30-2 HVF study was performed in each eye: (A) right eye and (B) left eye HVF: Humphrey visual field

The right visual field (shown in Figure 2A) showed dense, generalized constriction with a small area of preserved central vision. In comparison, the left visual field (shown in Figure 2B) was fully intact.

**Figure 3:**
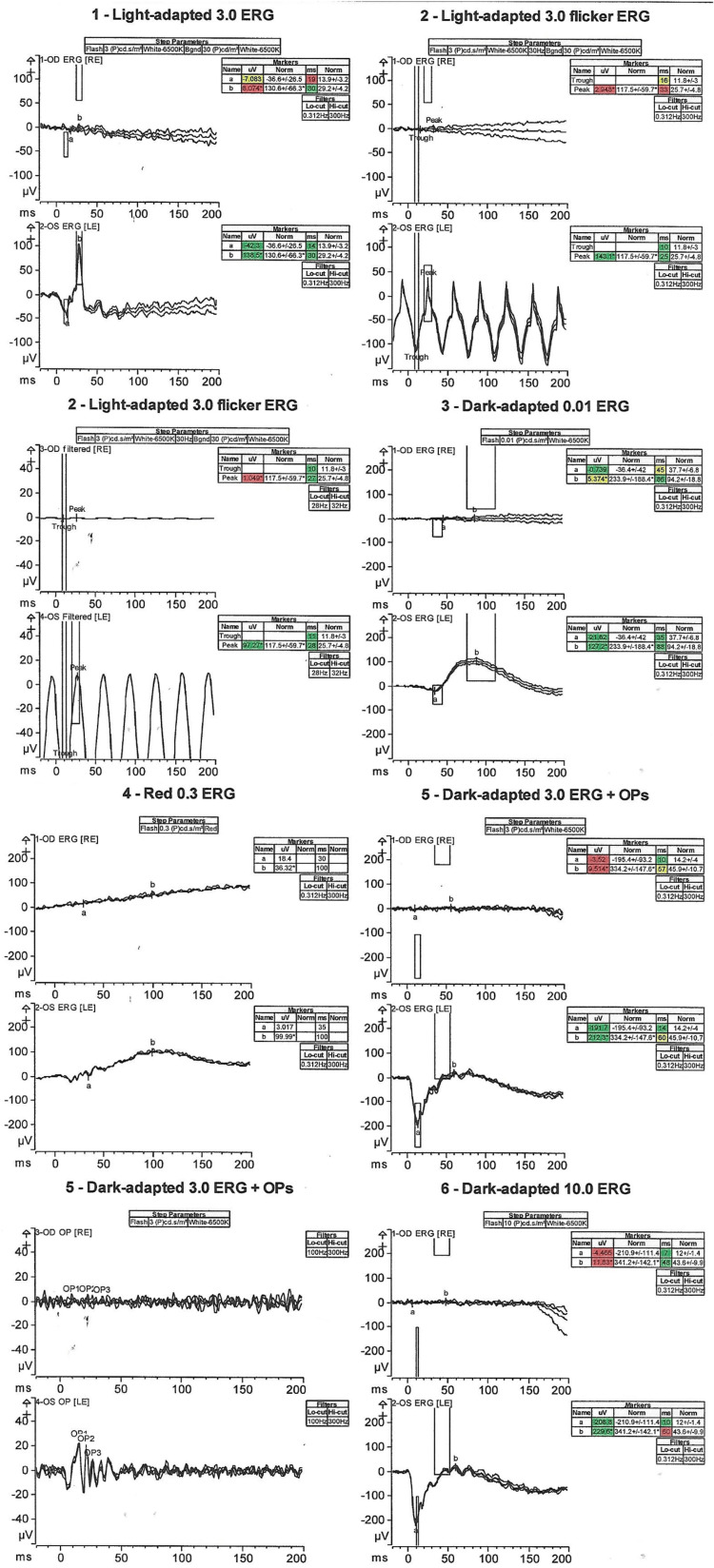
Electrical activity was measured by ffERG performed in each eye ffERG: full-field electroretinogram

ffERG showed decreased electrical signals in retinal cells of the right eye and abnormal a-wave and b-wave amplitudes (shown in Figure 3). While the ffERG revealed a nearly extinguished right eye response, electrical signaling in the left eye appeared to be stronger and largely within normal limits (shown in Figure 3). However, a discrepancy was observed in the dark-adapted ffERG, which showed abnormal patterning in the left eye, specifically a reduced b-wave that still remains within the standard deviation of normal.

**Figure 4:**
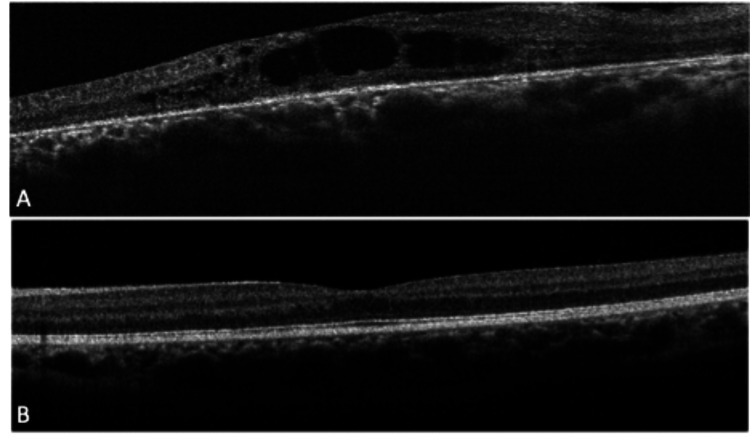
OCT of the macula in both eyes: (A) right eye and (B) left eye OCT: optical coherence tomography

Optical coherence tomography (OCT) of the right macula (shown in Figure 4A) showed moderate-to-severe, central CME within the outer plexiform layer and a blunted foveal contour. No medical treatment was initiated for the CME as it resolved spontaneously on subsequent visits. The right eye revealed subfoveal choroidal thickening and attenuated loss of the RPE layer. Comparatively, macula OCT of the left eye was within normal limits (shown in Figure 4B).

**Figure 5:**
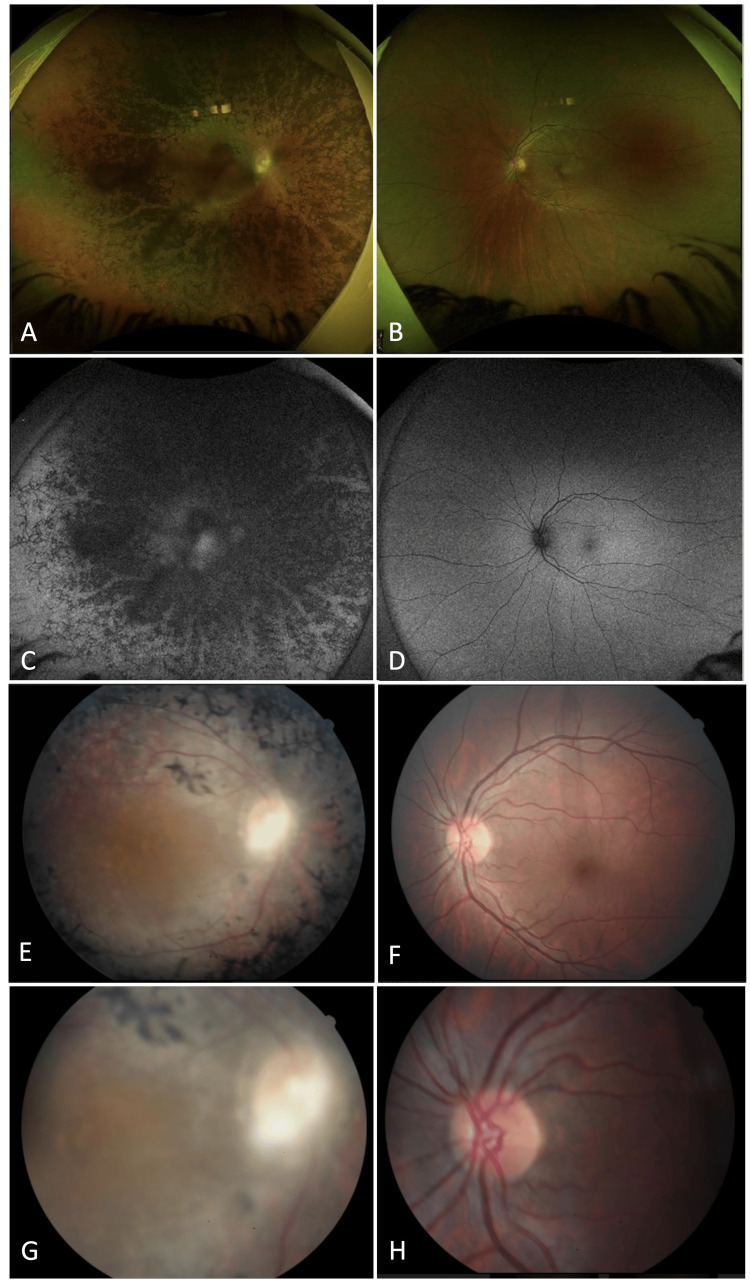
(A-D) Fundus autofluorescence of both eyes. (E-H) Fundus photography of both eyes

Indirect ophthalmoscopy, fundus photography, and fundus autofluorescence (FAF) of the right eye depicted extensive bone spicule pigmentation in the mid-peripheral to peripheral retina (shown in Figure 5A, 5C, 5E). Notably, no retinal drusen, hemorrhages, or exudates were observed, but arterial attenuation was appreciated in the right retina (shown in Figure 5A). Patchy and granular areas of autofluorescence were observed solely in the right eye (shown in Figure 5A, 5C). Fundoscopy of the right eye revealed significant waxy, optic nerve pallor and a crescent-shaped swath of glial tissue protruding from the nasal and inferior margins of the optic nerve (shown in Figure 5G). Fundoscopy and FAF of the left eye showed a healthy retina with well-perfused vasculature and unremarkable structural findings (shown in Figure 5B, 5D, 5F). The left optic nerve appeared healthy, with normal coloring and distinct disc margins (shown in Figure 5H).

Genetic testing was performed on a saliva sample utilizing a next-generation sequencing inherited retinal disease panel of 330 genes (Invitae, San Francisco, California, United States). A hybridization-based capture protocol was used to enrich DNA for targeted regions and subsequently sequenced on an Illumina platform. Reads were aligned to a reference sequence (GRCh37), and unless otherwise indicated, targeted regions were sequenced with ≥50 depth [13]. Analysis focused on the coding of relevant transcripts and up to 20 base pairs of flanking intronic sequence, but promoter regions, untranslated regions, and deep intronic sequences were not interrogated. Copy number variants, such as duplications or exonic deletions, were interpreted utilizing read-depth algorithms. Variants were interpreted and reported according to the Human Genome Variation Society (HGVS) nomenclature and classified based on the American College of Medical Genetics and Genomics (ACMG) and Association for Molecular Pathology (AMP) guidelines by Invitae geneticists. Orthogonal methods, including Sanger sequencing, were used to validate the reportable variants. Testing and confirmatory sequencing were performed by the Clinical Laboratory Improvement Amendments (CLIA)-certified and College of American Pathologists (CAP)-accredited Labcorp Genetics, Inc.

Genetic testing identified that the patient was heterozygous for the following variants (summarized in Table 1): INPP5E (c.1021G>A; p.Gly341Ser), FSCN2 (c.470G>C; p.Arg157Pro), RP1L1 (c.190C>T; p.Leu64Phe), and WFS1 (c.2266C>T; p.Arg756>Cys). 

**Table 1:** Salivary DNA testing showing four variants of variable interest for inherited retinal disorders from a panel of 330 genes

Gene	Variant	Zygosity	Variant classification
INPP5E	c.1021G>A (p.Gly341Ser)	Heterozygous	Pathogenic
FSCN2	c.470G>C (p.Arg157Pro)	Heterozygous	Uncertain significance
RP1L1	c.190C>T (p.Leu64Phe)	Heterozygous	Uncertain significance
WFS1	c.2266C>T (p.Arg756>Cys)	Heterozygous	Uncertain significance

## Discussion

This case showcases a unique presentation of URP and microphthalmia in a young patient. Examination of the right fundus showed peripheral bone spicule pigmentation typically associated with RP [1,3]. Likewise, patchy areas of decreased FAF were observed in the right eye, consistent with those of RP as reported in the literature [14]. The right eye visual field showed a severely constricted visual field, and ffERG depicted nearly extinguished retinal electrical activity, further supporting the RP diagnosis [1,3,6]. Although an incongruency was observed in the ffERG of the left eye, it could be explained by inadvertent motion due to feelings of anxiety, stress, or fatigue. Considering all other testing appeared within normal limits and the lack of symptomology in that eye, the unilateral manifestation of RP in this patient is still indicated [1,3,6]. Associated findings of a PSC cataract and CME in this patient in the right eye are also consistent with findings classic of RP patients [1]. Therefore, our patient meets all the criteria for diagnosis of URP as outlined previously. These changes have not manifested in the left eye since her original onset of symptoms [6]. While myelinated nerve fibers have been associated with other URP case reports [15], our patient's presentation of glial tissue protruding from the optic nerve does not appear to be myelinated nerve fibers, but instead some other form of glial tissue abnormality.

This patient presented with unilateral microphthalmia based on slit lamp exam and gonioscopy of the right eye. Additionally, the right eye's AL of 19.84 mm is more than two standard deviations shorter than the average eye [16]. Microphthalmia is often associated with abnormal retinal development which, coupled with photoreceptor degeneration, most likely contributes to the visual deficiencies in the right eye [16]. Although the AL of the left eye (22.06 mm) is within normal limits, it is shorter than average [16]. Gonioscopy revealed bilateral narrow anatomical chamber angles, with a more severe presentation in the right eye. IOP measurements, optic nerve exam, and Humphrey visual field (HVF) findings in both eyes suggest no evidence of glaucoma. However, her narrow anterior chamber anatomy warranted laser peripheral iridotomy in the right eye. This may also be performed in the left eye to prevent an acute angle-closure event.

Per patient report, she underwent ophthalmologic and genetic evaluation during childhood after failing a school vision screening. Although the original records were unavailable, the patient recalls being informed that the right eye may have had an isolated genetic abnormality. Salivary genomic testing performed in 2026 revealed a genetically sensitized retina as the patient is a heterozygote for the following variants: INPP5E (c.1021G>A; p.Gly341Ser), FSCN2 (c.470G>C; p.Arg157Pro), RP1L1 (c.190C>T; p.Leu64Phe), and WFS1 (c.2266C>T; p.Arg756>Cys). Of the four genes, the INPP5E variant is classified as pathogenic per the ACMG-AMP criteria and has been associated with AR RP and Joubert syndrome in a biallelic presentation [17]. INPP5E pathogenic variants disrupt phosphoinositide signaling, causing ciliary dysfunction, which, in turn, leads to photoreceptor degeneration [17]. However, our patient is only a carrier; therefore, this variant is insufficient to cause AR INPP5E-related disorders [17]. Comparatively, the FSCN2 gene does not have a strong established association with retinopathies, and not all variants in this gene cause pathogenic changes [18,19], underscoring the importance of this variant as of uncertain significance. Similar to the INPP5E gene, the RP1L1 gene encodes a component of the photoreceptor cilium, the axoneme [20]. RP1L1 variants have been associated with AD occult macular dystrophy (OCMD) and AR RP [20]. Despite its association with OCMD, the patient's normal visual acuity, HVF, and macular appearance in the left eye indicate that this variant does not explain the patient's phenotype. Therefore, the RPIL1 variant remains of uncertain significance. The WFS1 gene is associated with AR Wolfram syndrome and AD Wolfram-like syndrome [21]. Much like the variants in the FSCN2 and RP1L1 genes, not all genetic variants cause disease. The AD Wolfram-like syndrome typically presents with milder symptoms, commonly restricted to optic atrophy and sensorineural deafness, which was not observed in this patient, ruling out WFS1 variant involvement [21]. Despite the patient's genetically sensitized retinae, her genotype suggests she should be asymptomatic. Nevertheless, the localized degenerative retinal processes and ocular developmental abnormalities in the right eye indicate a potential postzygomatic mutation early in ocular development, such as in the right optic vesicle, either in the aforementioned genes or in another ciliary gene not detected in our testing. This allelic change in the right eye likely led to retinal ciliary dysfunction and the progression of her ocular phenotype. Of note, there have been rare cases of patients presenting with similar phenotypes of microphthalmia, RP, and optic nerve abnormalities related to mutations in the MFRP gene [22,23]; however, our patient's genetic results revealed variants of interest in the MFRP gene or the other observed symptoms of optic disc drusen or foveal hypoplasia.

## Conclusions

We report the simultaneous manifestation of URP, glial tissue abnormality, and microphthalmia in a young female patient with genetically sensitized retinae. The current case suggests a potential shared genetic profile between the three conditions, although causality cannot be determined. Future studies are required to better understand the pathogenesis of these potentially interrelated conditions, which, in turn, may lead to the development of experimental treatment modalities.
